# The Clinical Characteristics of Patients With Nonneutropenic Invasive Pulmonary Aspergillosis

**DOI:** 10.3389/fmed.2021.631461

**Published:** 2021-02-15

**Authors:** Lulu Liu, Yu Gu, Yu Wang, Kunlu Shen, Xin Su

**Affiliations:** ^1^Department of Respiratory and Critical Care Medicine, Jinling Hospital, Medical School of Nanjing University, Nanjing, China; ^2^Department of Respiratory and Critical Care Medicine, Jinling Hospital, Nanjing Medical University, Nanjing, China; ^3^Department of Respiratory and Critical Care Medicine, Jinling Hospital, Southern Medical University, Guangzhou, China

**Keywords:** invasive pulmonary aspergillosis, clinical manifestation, serum ferritin, serum GM, nonneutropenic

## Abstract

**Objective:** The goal of this study was to reveal the clinical manifestations of nonneutropenic invasive pulmonary aspergillosis (IPA), which are different from those of neutropenic patients.

**Methods:** The clinical data of patients with nonneutropenic IPA were collected at the Department of Respiratory and Critical Care Medicine, Jinling Hospital, from February 2009 to November 2019. We analyzed the general conditions, clinical manifestations, imaging findings, and laboratory tests of these IPA patients.

**Results:** A total of 116 patients with nonneutropenic IPA (31 proven and 85 probable) were included. They had an average age of 59.8 years. The most common underlying disease was chronic obstructive pulmonary disease (COPD, *n* = 33). The common clinical symptoms included cough (93.1%, *n* = 108), expectoration (59.5%, *n* = 69), fever (57.8%, *n* = 67), hemoptysis (30.2%, *n* = 35), and dyspnea (40.5%, *n* = 47). The common CT imaging manifestations included consolidation (47.4%, *n* = 55), cavities (47.4%, *n* = 55), air crescent sign (14.7%, *n* = 17), and nodules (8.6%, *n* = 10). Multiple lesions (74.1%, *n* = 86) were more common than single lesions (17.2%, *n* = 20) and diffuse lesions (8.6%, *n* = 10). The positive rate of laboratory tests was 88.2% (30/34) for BALF galactomannan (GM), 55.4% (56/101) for serum GM, 45.3% (48/106) for 1,3-β-D-glucan (BDG), 43.3% (46/106) for sputum culture, and 36.4% (20/55) for BALF culture. Patients who had high serum GM level [GM optical density index (ODI) >1] were more likely to have severe respiratory symptoms and higher serum ferritin. Further investigation showed that there was a positive correlation between serum GM level and serum ferritin level.

**Conclusion:** The clinical symptoms and radiological manifestations of nonneutropenic IPA are diverse and often lead to delayed diagnosis. It is important to become more vigilant of aspergillosis in nonneutropenic patients in order to achieve early diagnosis and treatment and to reduce mortality.

## Introduction

*Aspergillus* is a ubiquitous fungus that is abundant in nature air and is tiny enough (2–3 μm) to be inhaled into the small airway. *Aspergillus fumigatus* is the most common species of this genus, and it may cause invasive infection. The patients most at risk for invasive pulmonary aspergillosis (IPA) are neutropenic patients (1, 2). Due to the increasing prevalence of aging-related diseases and the widespread use of glucocorticoids and broad-spectrum antibiotics, nonneutropenic patients have experienced an increasing incidence of IPA. According to statistics from the American Healthcare Cost and Utilization Project—Nationwide Inpatient Sample database in 2004, the top three groups of patients most prone to invasive fungal infection (IFI) are those with chronic obstructive pulmonary disease (COPD), diabetes, and hematological malignancies (3). In contrast to patients with neutropenia, nonneutropenic patients have diverse clinical manifestations and often are ignored by clinicians for the consideration of IPA. So, they are easily misdiagnosed with other infectious diseases. Despite advances in both diagnosis and therapy, their mortality rate remains high. This article aims to explore the clinical features of IPA in nonneutropenic patients and provide valuable information for clinical diagnosis and treatment.

## Materials and Methods

Data from a total of 116 nonneutropenic IPA patients were collected from February 2009 to November 2019 at the Department of Respiratory and Critical Care Medicine, Nanjing Jinling Hospital. IPA was divided into proven and probable according to the EORTC/MSG criteria (4). Nonneutropenic: The absolute counts of peripheral blood neutrophils was >1.8^*^10^9^/L, which was consistent with nonneutropenia. Proven: Histopathology for *Aspergillus* was positive. Probable: There were dependable evidence of host factors, clinical manifestations, imaging findings on chest CT scan, and microbiological evidence [serum galactomannan (GM) or BALF GM, sputum culture]. We performed analysis of the general conditions, clinical manifestations, laboratory tests, and imaging features of 116 nonneutropenic IPA cases. Elevated serum ferritin was defined as values ≥1,000 μg/L.

Data are shown as median with interquartile range (IQR) for quantitative variables and as numbers (percentages) for qualitative variables. The Chi-square and Fisher's exact tests were used for categorical variables. An effect was considered to be statistically significant when the *P*-value was <0.05, and all significance tests were two tailed. The data were statistically analyzed using SPSS 22.0, and graphs were generated using GraphPad Prism.

## Results

### Patient Characteristics and Clinical Presentations

A total of 116 patients with nonneutropenic IPA were included in this study. Thirty-one cases were diagnosed as proven IPA by histopathologic evidence, while 85 cases were diagnosed as probable IPA. The demographic and clinical characteristics of nonneutropenic IPA are summarized in Table 1. The study population was 84 males and 32 females with an average age of 59.8 (19–84) years. COPD was the most common underlying disease among the patients (33 of 116 patients, 28.4%). A high rate of diabetes was also found (22 of 116 patients, 19%). The most common respiratory symptom was cough (108 cases, 93.1%) in these nonneutropenic IPA patients. Thirteen patients had received long-term (>2 weeks) glucocorticoid therapy.

**Table 1:** Characteristics of all 116 nonneutropenic invasive pulmonary aspergillosis (IPA) patients.

	**All IPA (*n* = 116)**
**BASELINE FACTORS**	
Women	32
Age, y	59.8 (19–84)
**UNDERLYING PULMONARY DISEASE**, ***N*** **(%)**	
Lung cancer	7 (6)
Bronchiectasis	9 (7.8)
Pulmonary tuberculosis	21 (18.1)
Chronic obstructive pulmonary disease (COPD)	33 (28.4)
**EXTRAPULMONARY DISEASE**, ***N*** **(%)**	
Diabetes	22 (19)
Autoimmune disease	6 (5.2)
Cardiovascular disease	27 (23.3)
**RESPIRATORY SYMPTOMS**, ***N*** **(%)**	
Cough	108 (93.1)
Expectoration	69 (59.5)
Fever	67 (57.8)
Hemoptysis	35 (30.2)
Dyspnea	47 (40.5)
**IMMUNOSUPPRESSIVE MEDICATION**, ***N*** **(%)**	
Long-term (>2 weeks) glucocorticoid	13 (11.2)

### Microbiological and Laboratory Findings

Detailed microbiological, laboratory, and CT thorax findings and are shown in Table 2. A serum GM optical density index (ODI) ≥ 0.5 was observed in 56 patients (55.4%), and a BALF GM ODI >0.7 (5) was observed in 30 patients (88.2%). The 1,3-β-D-glucan (BGD) test in serum samples had a lower positive rate (48 of 106 patients, 45.3%). Sputum *Aspergillus* cultures were performed in 106 patients, and 46 out of the 106 BALF cultures (43.3%) were positive for *Aspergillus*. The positive rate of BALF *Aspergillus* culture was 36.4% (20 of 55 patients). Histopathology was done in 45 patients, and the positive rate was 68.6%.

**Table 2:** The microbiological, laboratory, and CT thorax findings of 116 nonneutropenic IPA patients.

	**All IPA (*n* = 116)**
**LABORATORY FINDINGS**, ***N*** **(%)**	
IL-6, ng/L (45)	52.1 (10.08–266.5)
D-Dimer, mg/L (70)	1.24 (0.06–8.42)
Ferritin, μg/L (34)	544.80 (92.1–1,015.7)
CRP, mg/L (98)	71.31 (0.5–398.9)
ESR, mm/h (71)	54.49 (6–140)
Neutrophil, 10^9^/L (116)	9.63 (2.4–42.3)
**MYCOLOGICAL FINDINGS**, ***N*** **(%)**	
BALF galactomannan (GM) > 0.7 optical density index (ODI) (34)	30 (88.2)
Serum GM ≥ 0.5 ODI (101)	56 (55.4)
1,3-β-D-glucan (BDG) (106)	48 (45.3)
Sputum culture (106)	46 (43.3)
BALF culture (55)	20 (36.4)
Biopsy (45)	31 (68.9)
**CT THORAX FINDINGS**, ***N*** **(%)**	
Consolidation	55 (47.4)
Cavity	55 (47.4)
Ground-glass opacity	29 (25)
Nodule	10 (8.6)
Air crescent sign	17 (14.7)
Halo signs	4 (3.4)
Single lesion	20 (17.2)
Multiple lesions	86 (74.1)
Diffuse lesions	10 (8.6)

Clinical inflammatory-related indicators in IPA patients were above the normal values. In all patients, the median IL-6 concentration was 52.1 (10.08–266.5) ng/L, D-dimer was 1.24 (0.06–8.42) mg/L, CRP was 71.31 (0.5–398.9) mg/L, and ESR was 54.49 (6–140) mm/h. In addition to the inflammatory indicators, we also observed serum ferritin (median: 544.8 μg/L) above normal values in nonneutropenic IPA patients.

### The Relationship Between Serum GM Level and Clinical Factors

At present, the main clinical laboratory method used to diagnose pulmonary aspergillosis is serum GM testing. Therefore, we grouped the patients according to the serum GM level (<0.5 ODI vs. ≥0.5 ODI; ≤1 ODI vs. >1 ODI). Table 3 shows that patients with higher serum GM levels (GM >1 ODI) were more likely to have COPD and had more serious respiratory symptoms, hemoptysis, and dyspnea. In terms of imaging findings, patients with low serum GM (GM <0.5 ODI) had more single lesions, but multiple lesions were not necessarily more likely to appear in patients with high serum GM levels.

**Table 3:** Clinical factors associated with serum GM levels in nonneutropenic IPA patients.

	**Serum GM**		***P*-value**	**Serum GM**		***P*-value**
	**<0.5 ODI (*n* = 45)**	**≥0.5 ODI (*n* = 56)**		**≤1 ODI (*n* = 79)**	**>1 ODI (*n* = 22)**	
**BASELINE FACTORS**						
Women	13	15	0.814	22	6	0.957
Age, y	59.0 (19–84)	61.3 (21–84)	0.208	60 (19–84)	61.2 (32–84)	0.37
**UNDERLYING PULMONARY DISEASE**						
Lung cancer	5	2	0.138	6	1	0.618
Bronchiectasis	5	5	0.715	9	1	0.342
Pulmonary tuberculosis	11	6	0.067	14	3	0.651
COPD	13	19	0.588	28	4	0.05
**EXTRAPULMONARY DISEASE**						
Diabetes	9	12	0.86	15	6	0.397
Autoimmune disease	2	3	0.834	4	1	0.921
Cardiovascular disease	10	14	0.744	19	5	0.897
**RESPIRATORY SYMPTOMS**						
Cough	41	53	0.487	74	20	0.652
Expectoration	26	31	0.807	46	11	0.491
Fever	25	34	0.601	45	14	0.422
Hemoptysis	14	18	0.912	21	11	0.037
Dyspnea	25	42	0.04	57	10	0.019
**LABORATORY FINDINGS**						
IL-6, ng/L	31.0 (25)	78.6 (20)	0.005	48.8 (38)	70.2 (7)	0.371
D-Dimer, mg/L	1.4 (30)	1.1 (40)	0.288	1.3 (56)	1.0 (14)	0.395
Ferritin, μg/L	447.8 (14)	612.7 (20)	0.298	410.3 (27)	1,063.7 (7)	<0.0001
CRP, mg/L	60.4 (44)	80.2 (54)	0.136	68.6 (77)	81.3 (21)	0.432
ESR, mm/h	53.1 (29)	57.7 (34)	0.588	55.2 (50)	57.2 (13)	0.85
Neutrophil, 10^9^/L	8.2 (45)	9.6 (56)	0.26	9.1 (79)	8.5 (22)	0.71
**CT THORAX FINDINGS**						
Consolidation	18	30	0.175	35	13	0.219
Cavity	14	30	0.024	34	10	0.84
Nodule	5	5	0.715	8	2	0.886
Air crescent sign	3	9	0.147	11	1	0.229
Halo signs	2	2	0.823	3	1	0.847
Single lesion	11	5	0.034	12	4	0.734
Multiple lesions	32	44	0.388	60	16	0.757
Diffuse lesions	2	7	0.158	7	2	0.973

### Association of Elevated Ferritin With Serum GM

**Figure 1:**
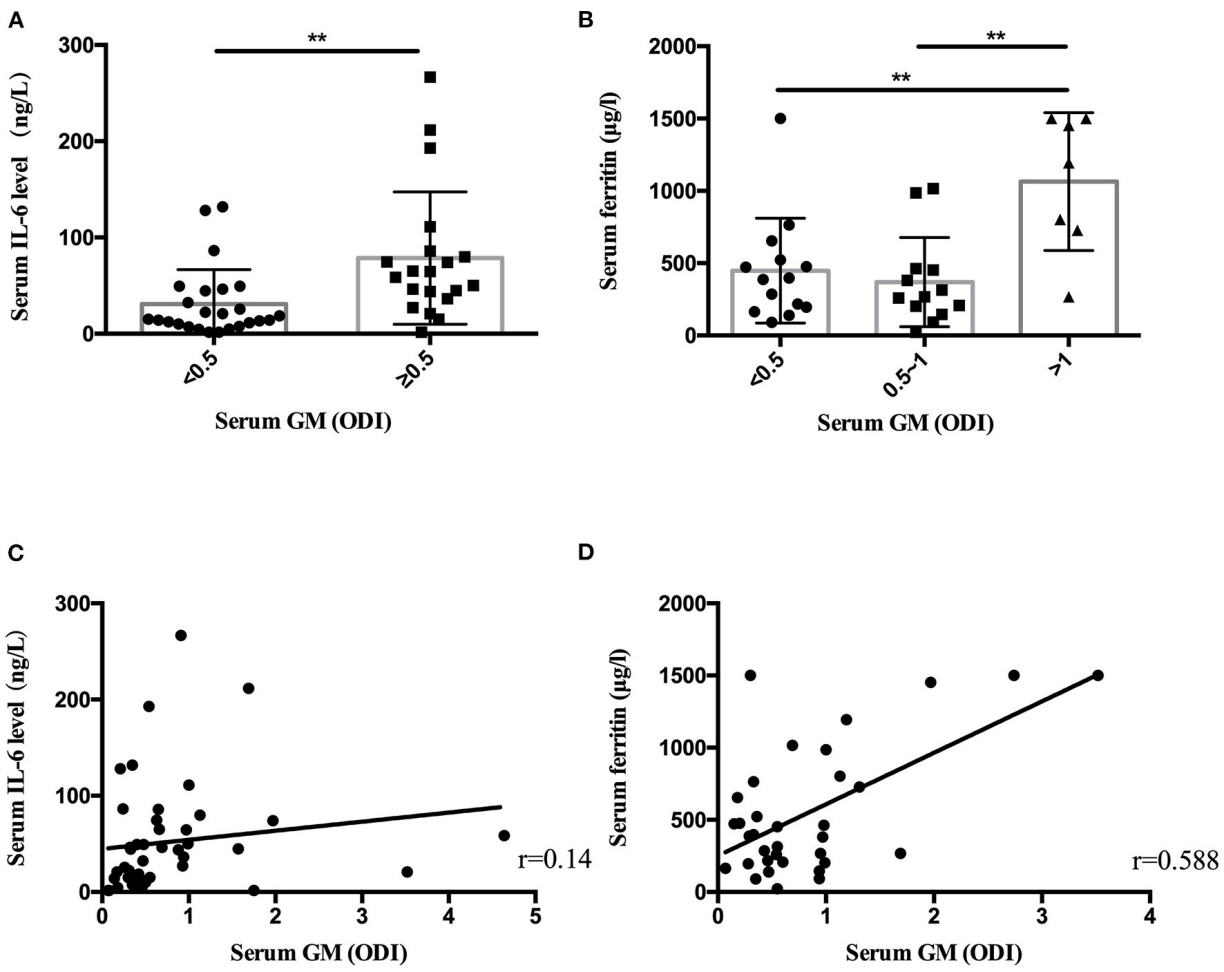
The serum IL-6 level and ferritin level in nonneutropenic invasive pulmonary aspergillosis (IPA). **(A)** The patients with serum galactomannan (GM) ≥ 0.5 optical density index (ODI) had higher serum IL-6 levels. **(B)** The patients with serum GM > 1 ODI had higher ferritin levels. **(C)** Scatter diagrams of serum GM vs. serum IL-6 level. **(D)** Scatter diagrams of serum GM vs. serum ferritin level. The value of *r* represents the correlation coefficients between the GM values and the IL-6 level and ferritin level. **P < 0.01.

Statistical analysis found that the patients with serum GM ≥0.5 ODI had higher serum IL-6, and the patients with serum GM >1 ODI had higher serum ferritin (Table 3). Further analysis revealed (Figure 1) that there was a positive correlation between serum GM level and serum ferritin level (*r* = 0.588, *P* = 0.0003), but serum GM had no significant correlation with IL-6 (*r* = 0.14, *P* = 0.358). Therefore, we compared the difference between the low-ferritin (<1,000 μg/L) and high-ferritin groups (≥1,000 μg/L) (6). Forty-two patients were tested for serum ferritin. Diabetes and expectoration were significantly more common in patients with high ferritin (Table 4).

**Table 4:** Differences in clinical characteristics according to serum ferritin.

**Characteristics**	**Ferritin <1,000 μg/L (*n* = 34)**	**Ferritin≥1,000 μg/L (*n* = 8)**	***P*-value**
**BASELINE FACTORS**			
Women	14	0	0.037
Age, y	60.9 (28–84)	66.4 (51–81)	0.151
**UNDERLYING DISEASE**			
Lung cancer	4	0	0.572
Autoimmune disease	3	0	1.0
Diabetes	4	4	0.03
Pulmonary tuberculosis	7	1	1.0
COPD	7	4	0.174
Bronchiectasis	5	2	0.601
Cardiovascular disease	7	4	0.174
**RESPIRATORY SYMPTOMS**			
Cough	32	8	1.0
Expectoration	20	8	0.037
Fever	16	5	0.697
Gasp	16	3	0.709
Hemoptysis	12	0	0.08
Dyspnea	9	1	0.655

## Discussion

IPA usually occurs in patients with hematological malignancies or patients who are severely immunocompromised (7). However, recent studies have found that the incidence of IPA is increasing year by year in nonneutropenic patients (8). The sample sizes of most studies of nonneutropenic IPA (9, 10) are small. This article included more nonneutropenic IPA patients and increased the reliability of our conclusion. A retrospective survey of pulmonary fungal epidemiology covering 16 studies in China (1997–2008) showed that, instead of hematological malignancies, the most common underlying diseases were solid tumors (14%), COPD (11%), tuberculosis (10%), and diabetes (10%) (11). Due to the large population and increasing morbidity, COPD is an increasingly common underlying disease in IPA patients, and the mortality rate of COPD with IPA is high (12, 13). We also found that COPD was the highest-incidence (33 of 116 patients, 28.4%) underlying disease in our nonneutropenic IPA patients.

Nonspecific clinical symptoms are one of the main causes of a delayed diagnosis of IPA. Fever is the most common clinical symptom in patients with neutropenic IPA (14). However, we found that the incidence of fever in nonneutropenic patients ranked third (57.8%), and the most common clinical symptom was cough (93.1%). In previous studies, it was found that the positive rate of halo signs was 95% in IPA patients with neutrophil deficiency, and the positive rate of air crescents was 33% (15). In this study, we found that consolidation (47.4%) and cavities (47.4%) were the most common imaging findings in nonneutropenic IPA patients, while typical halo signs and air crescent signs accounted for only 3.4 and 14.6%, respectively. Atypical clinical symptoms and chest CT imaging often lead to misdiagnosis of nonneutropenic IPA.

Biopsy is necessary to prove a diagnosis of pulmonary aspergillosis, but it is often difficult and risky to accomplish, especially in critically ill patients. Out of our 116 patients, only 45 patients underwent lung biopsy through percutaneous or transbronchoscopic lung puncture, and the positive rate was 68.9%. At present, *Aspergillus* antigen tests, such as the GM test, are common and reliable laboratory tests for the diagnosis of IPA. The positive rate of serum GM in our study was 55.4%; BALF GM had a higher positive rate (88.2%). Previous studies have also shown that the value of the BALF GM test was higher than that of the serum GM test in high-risk patients with IPA (16, 17). Although the positive rate of BAFL GM was higher, only 34 patients had the chance to be tested, because the bronchoscopy is not suitable for many critically ill patients. Respiratory cultures of *Aspergillus* from sputum (43.3%) and BALF (36.4%) had lower positive rates. Therefore, new diagnostic methods for detecting pulmonary *Aspergillus*, such as PCR and *Asp*LFD, are worth being carried out in clinical practice to help diagnose IPA (18–20).

We conducted a subgroup analysis to explore the relationship between GM level and different clinical manifestations. The results showed that patients with high serum GM level (>1 ODI) had more serious respiratory symptoms (hemoptysis and dyspnea), and patients with low GM level (<0.5 ODI) mainly showed a single lesion on thoracic CT imaging, suggesting that serum GM level might be related to the severity of the disease. Woods et al. (21) found a strong correlation between serum GM and aspergillosis outcome in both neutropenic and nonneutropenic patients. The survival of the patients whose serum GM normalized was significantly better than that of persistently positive patients. Other authors have confirmed that an increase in GM level at the time of diagnosis increases the risk of all-cause mortality at 6 weeks (22, 23).

In laboratory findings, we found that only the level of serum ferritin increased significantly in the high-GM group. Some small-sample-size studies showed increased ferritin levels or iron stores in patients who were diagnosed with invasive mold infections after allogeneic hematopoietic stem cell transplantation (24, 25). The decrease in GM values can be used to monitor the efficacy of a treatment (26). A recent study showed that elevated serum ferritin (>1,000 ng/ml) conferred an increased risk of fungal pulmonary infections (27). Our results suggest that serum ferritin was increased in nonneutropenic IPA patients (544.80 μg/L). In patients with serum GM >1 ODI, the ferritin level was significantly higher than in those patients with serum GM ≤1 ODI. We found that serum ferritin had a positive linear relationship with serum GM (*r* = 0.588, *P* = 0.000) in nonneutropenic IPA patients. In patients with elevated serum ferritin (≥1,000 μg/L), diabetes was more common (*P* = 0.03). Ford et al. (28) found that elevated serum ferritin levels were associated with insulin resistance and an increased risk of diabetes. The number of patients who received a ferritin test in our study was still small. Further research is necessary.

Taking our results together, we believe that the clinical symptoms and imaging manifestations of patients with nonneutropenic IPA are atypical. For patients with relevant underlying diseases and who failed to respond to antibiotic treatment, *Aspergillus* infection must be taken into consideration. Appropriate laboratory tests and prompt antifungal treatment are important to decrease the mortality.

## Data Availability Statement

The original contributions presented in the study are included in the article/supplementary material, further inquiries can be directed to the corresponding author/s.

## Ethics Statement

Ethical review and approval was not required for the study on human participants in accordance with the local legislation and institutional requirements. Written informed consent for participation was not required for this study in accordance with the national legislation and the institutional requirements.

## Author Contributions

XS and LL designed the study and drafted the manuscript. LL collected patients' data and analyzed the data. YG analyzed the data. YW and KS were critically involved in the data collection and the revision of the manuscript. All authors contributed to the article and approved the submitted version.

## Conflict of Interest

The authors declare that the research was conducted in the absence of any commercial or financial relationships that could be construed as a potential conflict of interest.
